# Musculoskeletal Digital Therapeutics and Digital Health Rehabilitation: A Global Paradigm Shift in Orthopedic Care

**DOI:** 10.3390/jcm14238467

**Published:** 2025-11-28

**Authors:** Youn Kyu Lee, Eun-Ji Yoon, Tae Hyung Kim, Jong-Ick Kim, Jong-Ho Kim

**Affiliations:** 1 School of Computer Science and Engineering, Chung-Ang University, Seoul 06974, Republic of Korea; 2 Department of Orthopaedic Surgery, Yeouido St. Mary’s Hospital, The Catholic University of Korea, Seoul 07345, Republic of Korea; 3 Department of Computer Engineering, Hongik University, Seoul 04066, Republic of Korea; taehyung@hongik.ac.kr; 4 Department of Orthopaedic Surgery, St. Onetop Hospital, Incheon 22145, Republic of Korea; moffats1@naver.com

**Keywords:** digital therapeutics, musculoskeletal rehabilitation, orthopedic surgery, tele-rehabilitation, artificial intelligence, wearable sensors, precision orthopedics

## Abstract

Musculoskeletal disorders (MSDs) affect over 1.7 billion people globally and represent the leading cause of disability worldwide. Conventional rehabilitation strategies face challenges including limited accessibility, suboptimal adherence, and lack of personalization. Digital therapeutics (DTx)—evidence-based, software-driven interventions regulated as medical devices—have emerged as transformative solutions in chronic disease management. This review provides a narrative synthesis of representative studies in the field, drawing on a broad survey of literature from medical and engineering sources to capture current trends and clinically relevant developments. Seventy-five publications were examined, including clinical trials and validation studies, many of which reported outcomes comparable or superior to traditional rehabilitation approaches, with adherence gains of 15–40% and cost reductions of approximately 30–40%. We summarize the major technological foundations of musculoskeletal DTx and digital rehabilitation across orthopedic subspecialties, describing core-enabling technologies including artificial intelligence-driven motion analysis, wearable sensors, tele-rehabilitation platforms, and cloud-based ecosystems. Clinical applications spanning spine, upper and lower extremities, sports injuries, and trauma were analyzed alongside global regulatory frameworks, economic considerations, and implementation challenges. Early clinical evidence demonstrates improvements in functional outcomes, adherence, and cost-effectiveness. Future directions include digital twin-based precision rehabilitation, predictive analytics, and scalable integration into value-based orthopedic care. By establishing a comprehensive framework for musculoskeletal DTx implementation, this review highlights their potential to improve outcomes, reduce healthcare costs, and address global rehabilitation access gaps. However, evidence on long-term effectiveness, sustained cost benefits, and large-scale clinical integration remains limited and warrants further investigation.

## 1. Introduction

### 1.1. Epidemiology and Global Burden of Musculoskeletal Disorders

Musculoskeletal disorders (MSDs) constitute a diverse group of conditions affecting muscles, bones, tendons, ligaments, and joints, ranging from degenerative diseases to traumatic injuries and chronic pain syndromes [1,2]. The Global Burden of Disease study identifies MSDs as the leading contributor to disability-adjusted life years worldwide, affecting approximately 1.71 billion individuals [3]. This staggering burden encompasses a wide spectrum of conditions including osteoarthritis affecting 528 million people globally, low back pain experienced by 619 million individuals, and rheumatoid arthritis impacting 18 million patients worldwide [2]. The economic impact is equally substantial, with MSDs accounting for an estimated $213 billion in annual healthcare expenditures in the United States alone and representing the second highest category of healthcare spending after cardiovascular disease [3].

Paradoxically, increased participation in recreational sports activities among both younger and older populations has led to higher rates of sports-related musculoskeletal injuries. Additionally, the COVID-19 pandemic has resulted in a substantial backlog of elective orthopedic procedures, with an estimated 28 million surgeries worldwide deferred during 2020–2021, creating unprecedented rehabilitation demands [4].

### 1.2. Evolution of Orthopedic Surgical Practice and Rehabilitation Needs

Orthopedic surgical volumes are increasing steadily across all major procedure categories. Total knee arthroplasty (TKA) and total hip arthroplasty (THA) are projected to grow by 85–284% over the next decade, with TKA procedures alone expected to reach 3.5 million annually in the United States by 2030 [5,6]. From 2007 to 2013, shoulder arthroscopy procedures performed per resident increased by 43.1% in the U.S.A [7]. This demographic shift necessitates innovative approaches to perioperative care and rehabilitation that can scale to meet demand without proportionally increasing healthcare workforce requirements or infrastructure costs.

Rehabilitation remains essential for optimizing outcomes in both postoperative recovery and chronic condition management [8]. Evidence consistently demonstrates that high-quality, intensive rehabilitation programs improve functional outcomes, reduce complication rates, and enhance patient satisfaction following orthopedic procedures. For example, early high-intensity rehabilitation following TKA has been shown to improve strength by 40%, functional performance by 35%, and quality of life measures by 30% compared to standard care. However, conventional rehabilitation models face critical limitations that compromise their effectiveness and accessibility [9].

### 1.3. Limitations of Conventional Rehabilitation Models

Traditional rehabilitation approaches encounter multiple systemic barriers that limit their effectiveness and reach. Geographic barriers restrict access to physiotherapy services, particularly in rural and underserved areas where over 20% of the US population resides but only 9% of physical therapists practice. Socioeconomic factors compound these challenges, with lower-income patients demonstrating 40% lower rehabilitation attendance rates due to transportation costs, time constraints related to employment, and limited health insurance coverage for extended therapy sessions [10].

Patient adherence to prescribed home exercise programs remains disappointingly low across all musculoskeletal conditions, with compliance rates ranging from 30 to 65% in various studies [11,12]. Multiple factors contribute to poor adherence including lack of supervision and feedback, difficulty remembering exercise protocols, insufficient motivation, fear of pain or reinjury, and absence of objective progress tracking [13,14]. This adherence gap directly impacts clinical outcomes, with non-adherent patients demonstrating 50% worse functional recovery and 70% higher complication rates compared to adherent individuals.

Recent evidence indicates that digital follow-up and telerehabilitation platforms markedly improve adherence through continuous monitoring and real-time feedback. A 2022 systematic review and meta-analysis reported mean adherence rates ranging from 65 to 85%, with digital rehabilitation compared to 40–60% in conventional programs [15]. Similarly, a 2024 meta-analysis of randomized controlled trials in total knee arthroplasty patients found equivalent functional outcomes but significantly higher completion and satisfaction rates with telerehabilitation groups [16]. These findings highlight the potential of digital therapeutics to overcome one of the key limitations of conventional rehabilitation-poor engagement and adherence between clinic visits.

Furthermore, traditional rehabilitation models rely heavily on intermittent, subjective assessments during periodic clinic visits, typically occurring weekly or biweekly [17]. This episodic monitoring approach provides limited insight into patient behavior during the critical intervals between appointments and delays identification of complications or adherence issues. Clinicians lack real-time data on exercise performance quality, cannot dynamically adjust protocols based on daily progress, and have limited ability to predict patients at risk for poor outcomes. The subjective nature of many clinical assessments introduces inter-rater variability and makes it challenging to establish objective benchmarks for progression criteria.

### 1.4. Emergence of Digital Therapeutics as a Solution

Digital therapeutics (DTx) represent a distinct category of regulated, evidence-based software interventions designed to treat, manage, or prevent medical conditions [18,19]. Unlike general wellness applications or fitness trackers, DTx undergo rigorous clinical validation and regulatory approval processes analogous to pharmaceuticals or implantable devices [20]. The Digital Therapeutics Alliance defines DTx as products that deliver medical interventions directly to patients using evidence-based, clinically evaluated software to treat, manage, or prevent a medical disorder or disease [21]. Importantly, DTx are classified as Software as a Medical Devise (SaMD) under regulatory frameworks such as the FDA and European MDR when they directly deliver a therapeutic function or influence clinical outcomes. In contrast, digital support tools that provide education, monitoring, or motivation without a therapeutic indication are not subject to such regulatory oversight and do not meet DTx criteria [22,23]. More broadly, DTx represent a regulated subset of SaMD with explicit therapeutic intent, whereas digital support tools and general health applications lack therapeutic claims, which are not subject to medical-device regulatory pathways, and primarily function to provide education, tracking, or motivation rather than deliver a medical intervention.

The evolution of DTx has been enabled by convergence of multiple technological advances over the past decade. Smartphone penetration has reached 85% in developed countries and over 60% globally, providing ubiquitous access to computing power previously confined to specialized laboratories [24]. Cloud computing infrastructure has enabled scalable, secure data storage and processing capabilities. Artificial intelligence (AI) and machine learning algorithms have matured to enable sophisticated pattern recognition and predictive analytics. Wearable sensor technology has miniaturized while improving accuracy and battery life. High-speed wireless connectivity through 4G and 5G networks enables real-time data transmission and remote monitoring [25].

By leveraging these enabling technologies, musculoskeletal DTx can provide personalized, adaptive rehabilitation protocols with continuous, objective monitoring of patient progress [26,27]. These systems extend rehabilitation beyond clinic walls into patients’ homes and daily environments, enhance patient engagement through gamification, social support, and real-time feedback mechanisms, and generate rich datasets for predictive analytics and continuous protocol optimization [28,29]. Despite these opportunities and growing evidence of effectiveness in other medical specialties, musculoskeletal DTx remain in relatively early developmental stages with significant potential for expansion and refinement [30]. It is also important to note that the technological maturity of musculoskeletal DTx varies widely across categories: telerehabilitation platforms are supported by well-established clinical trials, wearable-sensor systems and computer-vision tools have demonstrated intermediate levels of validation, and AI-driven analytics, predictive models, and digital biomarkers remain in comparatively earlier developmental stages. However, the field continues to face important limitations, including heterogeneous levels of evidence across technologies, uncertainties regarding optimal clinical integration pathways, and ongoing debate surrounding the regulatory distinction between therapeutic and non-therapeutic digital health software.

### 1.5. Objectives and Scope of This Review

This review provides a narrative synthesis of the current landscape of musculoskeletal digital therapeutics across postoperative orthopedic rehabilitation and chronic musculoskeletal conditions. The purpose of this work is to integrate key technological, clinical, and practical themes rather than to conduct a protocol-driven or systematic evidence assessment. By reviewing representative and influential studies, this review aims to offer a clinically meaningful overview and framework for the integration of digital therapeutics in orthopedic care.

The specific objectives are as follows:

(1) Describe the core enabling technologies—such as AI, wearable sensors, cloud-based platforms, and immersive interfaces—that underpin musculoskeletal DTx;
(2) Examine clinical applications and evidence across orthopedic subspecialties (shoulder, spine, knee, and sports medicine);
(3) Analyze global regulatory frameworks, reimbursement models, and implementation challenges;
(4) Identify emerging directions such as digital twin technologies, precision rehabilitation, and global scalability strategies;
(5) Provide practical guidance for clinicians, healthcare systems, and developers integrating musculoskeletal DTx into clinical practice.

In addition, this review extends prior work by synthesizing evidence across multiple musculoskeletal subspecialties and integrating technological, regulatory, and implementation perspectives, thereby providing a broader and more clinically oriented overview than earlier domain-specific reviews.

## 2. Methods

This review was developed as a narrative synthesis to summarize current evidence on musculoskeletal digital therapeutics and technology-assisted rehabilitation. A broad survey of the literature across medical and engineering fields was performed to identify representative and influential studies. No prespecified protocol, PRISMA framework, or formal screening steps were applied. Instead, articles were selected for their relevance to digital therapeutic technologies, clinical applicability, and emerging trends.

The available literature demonstrated substantial methodological heterogeneity that precluded any meaningful quantitative pooling. Included studies varied widely in design (from randomized controlled trials to feasibility and technology-development studies), target populations, anatomical regions, intervention types (e.g., telerehabilitation platforms, wearable sensors, computer-vision systems, AI-based models, VR programs), comparator treatments, outcome measures, and follow-up durations. In addition, many technology-focused publications reported validation or performance metrics rather than clinical endpoints, making them incompatible with standardized effect-size calculations. Representative digital rehabilitation solutions referenced in the included studies encompassed commercially available platforms such as Sword Health (Porto, Portugal; Salt Lake City, UT, USA), Hinge Health (San Francisco, CA, USA), AppliedVR (Los Angeles, CA, USA), FeetMe smart insoles (Paris, France), and VERA^TM^ (Reflexion Health, San Diego, CA, USA), as well as commonly used VR devices including Oculus Quest (Meta Platforms, Inc., Menlo Park, CA, USA) and HTC Vive (HTC Corp., Taoyuan, Taiwan). For these reasons, a narrative, descriptive synthesis was the most appropriate approach for integrating findings across such diverse evidence streams.

### 2.1. Search Strategy and Information Sources

A broad literature search was conducted across major electronic databases, including PubMed/MEDLINE, Embase, Web of Science, Cochrane Library, and IEEE Xplore from inception through April 2025, to capture relevant studies on musculoskeletal digital therapeutics and technology-assisted rehabilitation. The search strategy employed a combination of controlled vocabulary terms and free-text keywords related to digital therapeutics, musculoskeletal rehabilitation, telemedicine, AI, and specific orthopedic subspecialties. Representative examples of search terms included (“digital therapeutic*” OR “digital health” OR “mHealth” OR “eHealth” OR “telerehabilitation” OR “tele-rehabilitation” OR “virtual rehabilitation” OR “remote monitoring”) AND (“musculoskeletal” OR “orthopedic” OR “physiotherapy” OR “physical therapy” OR “rehabilitation”) AND (“artificial intelligence” OR “machine learning” OR “wearable sensor” OR “mobile application*” OR “smartphone”).

Additional information sources included regulatory agency documents (FDA, EMA, PMDA), ClinicalTrials.gov, conference proceedings from major orthopedic societies (AAOS, EFORT, ISAKOS), and gray literature such as industry white papers and health technology assessment reports. Reference lists of key publications were also reviewed to identify related work, and influential articles were used to follow recent developments in the field.

Because this review was designed as a narrative synthesis, the search process focused on gathering influential and conceptually relevant studies rather than implementing a protocol-based or formally structured screening procedure. Search strategies were adapted for each database, and studies were selected based on thematic relevance to musculoskeletal digital therapeutics, technological innovation, and clinical applicability. No formalized PRISMA-style screening steps or quantitative synthesis procedures were applied.

Representative examples of the search terminology used across databases are included in Appendix A to offer additional context regarding the literature search approach.

### 2.2. Inclusion and Exclusion Criteria

This review focused on publications describing digital therapeutics, digital health interventions, and technology-enabled rehabilitation approaches relevant to musculoskeletal conditions. Studies were considered when they involved human participants or presented technologies intended for clinical use and were published in English. Sources that contributed original clinical data, technological development, regulatory perspectives, or expert consensus were all included within the scope of the review. Both clinical research such as randomized trials, cohort studies, and case series and descriptive or operational studies, including technology descriptions, implementation analyses, and economic evaluations, were examined to provide a broad understanding of current practice.

Work that centered exclusively on diagnostic imaging or surgical-planning tools without a rehabilitation component, general wellness or fitness applications without therapeutic intent or clinical validation, non-musculoskeletal conditions, or abstract-only reports without accessible full text was not emphasized in the narrative synthesis.

Rather than applying formal selection criteria, studies were incorporated based on their thematic relevance to musculoskeletal digital therapeutics, clinical applicability, and contribution to emerging technological or implementation trends. Although older foundational work was referenced when informative, particular attention was given to publications from the past five years due to the rapid pace of technological advancement. A quantitative summary of the number of records identified or screened is not presented, as this review does not follow a protocol-driven systematic methodology.

Clinical domains primarily emphasized shoulder and upper extremity, spine, and lower extremity rehabilitation, as these represent the major anatomical regions with the most mature evidence base in digital therapeutics and validated outcome measures. Other musculoskeletal areas (e.g., hip, hand, or trauma-related rehabilitation) were also reviewed when relevant, although peer-reviewed data remain limited and standardized digital assessment tools are less established.

### 2.3. Data Extraction and Synthesis

Data from the selected publications was organized using a structured summary format that captured study design and population characteristics, intervention details including technological components and delivery mechanisms, comparator interventions, reported outcomes, implementation considerations, and regulatory or economic information. For clinical studies, particular attention was paid to functional outcome measures, adherence rates, safety outcomes, and cost-effectiveness data.

Given the heterogeneity of included studies in terms of design, populations, interventions, and outcomes, a narrative synthesis approach was employed rather than quantitative meta-analysis. Studies were organized thematically by technology type, clinical application area, and implementation domain. Where multiple studies addressed similar questions with comparable methodologies, findings were synthesized to identify consistent patterns and areas of uncertainty.

This review did not apply formal risk-of-bias scoring frameworks, as the purpose was to provide a descriptive and interpretive synthesis rather than a protocol-driven methodological evaluation. However, methodological aspects reported within the original studies such as blinding practices, sample size, and duration of follow-up were considered when discussing the robustness of their findings. The key characteristics of representative clinical trials included in the review are summarized in Table 1 to illustrate the range of study designs, technologies, and outcomes reported.

### 2.4. Regulatory and Economic Framework Analysis

Regulatory considerations were examined by reviewing official guidance documents, approval pathways, and case studies from major regulatory agencies including the US Food and Drug Administration (FDA), European Medicines Agency (EMA), Japan’s Pharmaceuticals and Medical Devices Agency (PMDA), and Korea’s Ministry of Food and Drug Safety (MFDS). Regulatory documents were identified through a manual review of official agency websites and publicly available databases, including FDA guidance repositories, EMA and PMDA publications, and MFDS notices. To ensure that key regulatory perspectives were captured, these sources were supplemented by regulatory frameworks referenced within academic publications and industry reports. Economic perspectives were informed by reviewing health technology assessment documents, cost-effectiveness studies, and reimbursement policy documents from various healthcare systems.

## 3. Core Technologies Underpinning Musculoskeletal DTx

### 3.1. Motion Analysis and Computer Vision

Recent advances in computer vision and machine learning have enabled markerless motion capture using standard smartphone cameras or low-cost sensors, allowing quantitative movement analysis outside laboratory environments [39,40,41].

These technologies employ pose estimation and biomechanical modeling algorithms to calculate joint angles and movement quality with accuracy comparable to traditional goniometry [42,43,44]. Reported measurement reliability typically ranges from good to excellent (intraclass correlation coefficient, ICC > 0.85), indicating clinically acceptable accuracy for joint angle assessment in upper and lower extremities.

Clinical validation studies demonstrate that smartphone- or camera-based systems achieve excellent reliability and validity for shoulder, hip, and lower-limb joint assessments, supporting their integration into remote rehabilitation and telemedicine workflows [42,43,44]. Such systems can provide automated feedback and enable real-time monitoring of exercise performance, potentially improving adherence and facilitating personalized rehabilitation.

Recent developments incorporating AI-driven motion recognition and multi-view imaging have further enhanced exercise classification accuracy and movement-quality scoring, approaching expert physiotherapist performance levels [45,46]. These advances indicate that computer vision-based motion analysis is now sufficiently mature to complement or even replace traditional laboratory-based systems in musculoskeletal rehabilitation practice.

Detailed descriptions of algorithmic architectures, computational pipelines, and validation metrics are provided in Appendix B (Table A1).

### 3.2. Wearable Sensors and Continuous Monitoring

Wearable devices provide continuous, unobtrusive tracking of physical activity and physiological signals during rehabilitation and daily life, offering objective and longitudinal data on musculoskeletal function. [47,48,49].

Among various modalities, inertial measurement units (IMUs) embedded in wearable devices such as smart bands, patches, clothing, and insoles are the most widely used. These multi-sensor systems capture motion dynamics including joint angles, acceleration, and loading patterns with high temporal resolution, supporting accurate quantification of rehabilitation performance [48,49].

Electromyography (EMG) sensors complement motion data by monitoring muscle activation patterns and fatigue, enabling biofeedback-based training and assessment of neuromuscular recovery [50,51,52]. Similarly, smart insole systems capture real-time gait characteristics and weight-bearing patterns, providing clinicians with feedback on adherence to rehabilitation protocols, especially after lower extremity surgery [53,54].

Upper extremity wearable systems, including smart watches, arm bands, and finger-mounted sensors, provide objective measures of limb use, range of motion, and movement frequency, during daily activities [55]. These systems address the critical challenge of monitoring actual limb use rather than merely capacity for use, distinguishing between compensatory strategies and true functional recovery. For example, following rotator cuff repair or distal radius fracture, wearable systems can quantify actual arm elevation frequency and duration throughout the day, providing objective metrics that complement traditional clinic-based assessments. Detailed technical specifications, and validation outcomes for each wearable sensor modality are summarized in Appendix B (Table A2).

### 3.3. Cloud-Based Platforms and Artificial Intelligence

Cloud-based architectures form the digital backbone of musculoskeletal digital therapeutics, enabling continuous and secure data exchange between wearable devices, patient interfaces, and clinicians [56,57]. These systems integrate edge computing, encrypted data transmission, and cloud storage to ensure efficient, privacy-preserving, and reliable information management with 99.99% service availability and redundancy [57,58]. Interoperability with electronic health record systems via standardized application programming interfaces (APIs) enables seamless integration into clinical workflows and bidirectional exchange of rehabilitation data [57].

Clinician dashboards provide intuitive, multi-stream visualizations of patient adherence, range of motion, pain trends, and outcome metrics, supporting remote decision-making and early detection of adverse patterns.

AI-driven analytics enhance these cloud systems by transforming multimodal sensor data into personalized and adaptive rehabilitation pathways [59,60,61,62]. Machine learning models—including supervised, reinforcement, and deep neural networks—predict functional recovery trajectories and dynamically optimize exercise intensity [61,62,63]. Adaptive algorithms automatically adjust exercise parameters based on real-time performance and symptoms, yielding 25–40% improvements in functional outcomes compared with static protocols [63]. Predictive models trained on large clinical datasets can identify patients at high risk of postoperative complications such as stiffness or retear, with reported achieve areas under the receiver operating characteristic curve (AUC-ROC) of 0.75–0.88 for rotator cuff retear prediction [64]. Emerging digital-twin frameworks integrate biomechanical, surgical, and physiological data to simulate individualized rehabilitation scenarios, allowing clinicians to test and refine intervention strategies before implementation [65,66]. A consolidated overview of system architectures, AI model types, and reported validation findings is provided in Appendix B (Table A3).

### 3.4. Virtual and Augmented Reality

Virtual and augmented reality (VR/AR) technologies are increasingly incorporated into musculoskeletal DTx to enhance engagement, motivation, and motor learning through immersive experiences [67,68]. These technologies leverage principles of neuroplasticity and motor learning theory, using multisensory feedback and ecological validity to accelerate skill acquisition and functional recovery.

Clinical evidence shows that VR-based rehabilitation improves exercise adherence by 40–60% compared with conventional programs, driven by enhanced engagement and real-time feedback [69,70].

VR environments immerse patients in gamified or task-oriented exercises, while AR overlays guidance cues such as optimal movement trajectories or joint alignment feedback onto the patient’s real surroundings [31,71].

Beyond functional rehabilitation, VR has proven efficacy in pain management, reducing acute pain perception by 30–50% and achieving medium-to-large effect sizes (Cohen’s d = 0.5–0.8) for chronic musculoskeletal pain relief [72,73].

Recent implementations combine haptic feedback, biofeedback integration, and networked “social VR” therapy sessions to promote adherence and shared rehabilitation experiences [74]. Technical optimization (latency < 20 ms, refresh rate > 90 fps) and accessibility design remain essential for clinical safety and usability. A summary of key VR/AR hardware characteristics, therapeutic approaches, and reported performance findings is provided in Appendix B (Table A4).

## 4. Clinical Applications Across Orthopedic Subspecialties

Across the musculoskeletal domains reviewed, this narrative synthesis draws on over 20 clinical and observational studies directly evaluating digital therapeutics or technology-enabled rehabilitation, including more than 1200 participants enrolled in randomized or controlled trials and prospective cohorts. The most nature evidence bases are found in postoperative rotator cuff repair and total knee arthroplasty, where multiple controlled trials with sample sizes in the hundreds demonstrate non-inferior or improved outcomes compared with conventional in-person rehabilitation. In contrast, applications in anterior cruciate ligament reconstruction, adolescent idiopathic scoliosis, and fracture care are supported primarily by single-center randomized trials, pilot studies, and early-phase cohorts with tens to low hundreds of patients, reflecting earlier stages of clinical validation in these sub-areas.

### 4.1. Shoulder and Upper Extremity

Digitally assisted rehabilitation after arthroscopic rotator cuff repair (ARCR) has demonstrated comparable or improved outcomes relative to conventional therapy in several published randomized controlled trials. A landmark study by Correia et al. randomized 50 patients to either sensor-guided home rehabilitation or conventional physiotherapy following ARCR [31]. The digital intervention utilized wearable IMU sensors integrated with a mobile application providing real-time feedback on exercise performance. At 12 weeks, both groups achieved similar early functional outcomes with no significant differences in Constant-Murley Score (82.4 vs. 80.1, *p* = 0.34) or range of motion measurements. However, at 12-month follow-up, the digital rehabilitation group demonstrated significantly better QuickDASH scores (8.2 vs. 14.7, *p* = 0.019) and Constant-Murley scores (89.3 vs. 83.6, *p* = 0.027), representing clinically meaningful improvements. Importantly, the digital intervention reduced resource utilization by 40%, with patients requiring an average of 3.2 in-person visits compared to 12.4 in the conventional group, generating estimated cost savings of $1800 per patient.

Another assessor-blinded, randomized controlled trial by Shim et al. enrolled 105 patients and compared augmented reality-based digital rehabilitation to standard rehabilitation following ARCR [32]. The digital system provided visual overlays guiding patients through precise movement trajectories and offering real-time corrective feedback. At 3 months postoperatively, the digital rehabilitation group achieved superior outcomes across multiple domains: mean forward flexion improved to 156° versus 142° in controls (*p* = 0.003), external rotation at the side reached 52° versus 46° (*p* = 0.018), and pain VAS scores were significantly lower (2.1 vs. 3.4, *p* = 0.007). Patient satisfaction scores were notably higher in the digital group (8.7/10 vs. 7.2/10, *p* < 0.001), with qualitative feedback highlighting appreciation for the detailed guidance and ability to verify correct exercise performance independently. Adherence rates measured through app logging showed 81% of prescribed exercises completed in the digital group compared to estimated 58% compliance in the control group based on patient diaries (*p* < 0.001).

Telehealth implementations for postoperative follow-up have also proven effective. Kane et al. conducted a prospective randomized trial demonstrating that telehealth follow-up visits were non-inferior to in-person visits for monitoring recovery after rotator cuff repair [75]. Patient satisfaction was equivalent between groups (89% vs. 91%, *p* = 0.67), no adverse events or complications were missed during telehealth visits, and the remote model provided substantial convenience benefits with patients saving an average of 3.2 h and $85 in travel costs per visit.

### 4.2. Spine Care

#### 4.2.1. Chronic Low Back Pain

Chronic low back pain (CLBP) represents one of the most extensively researched indications for digital therapeutics, given its high prevalence (affecting 7.5% of the global population), substantial disability burden, and evidence supporting multidisciplinary biopsychosocial interventions that are resource-intensive to deliver through traditional models [76].

A landmark preregistered randomized controlled trial by Shebib et al. evaluated a comprehensive 12-week multimodal digital care program in 140 patients with CLBP [33]. The intervention integrated multiple components: sensor-guided exercise therapy using motion capture for real-time form correction and progression, educational modules addressing pain neuroscience and self-management strategies, cognitive behavioral therapy exercises for psychological factors, and remote coaching from licensed physical therapists providing weekly guidance and motivation. The control group received usual care consisting of education materials and physician-recommended exercises without technological support.

Results demonstrated significant improvements favoring digital intervention. Pain scores on the Numeric Rating Scale decreased by 3.2 points in the digital group compared to 1.1 points in usual care (between-group difference −2.1, 95% CI: −2.8 to −1.4, *p* < 0.001), exceeding the minimal clinically important difference of 2 points. Functional disability measured by the Oswestry Disability Index improved by 12.5 points versus 4.3 points in controls (between-group difference −8.2, 95% CI: −11.4 to −5.0, *p* < 0.001). Adherence was notably high, with 82% of digital group participants completing ≥80% of prescribed exercises compared to estimated 45–50% adherence in usual care based on historical controls. Healthcare utilization patterns shifted favorably, with the digital group demonstrating 35% fewer emergency department visits, 28% fewer imaging studies, and 40% lower opioid prescription rates at 12-month follow-up.

Virtual reality therapeutics have emerged as a promising modality for CLBP management. Garcia et al. conducted a double-blind randomized controlled trial of RelieVRx, an at-home VR program incorporating cognitive behavioral therapy, mindfulness, pain education, and relaxation techniques delivered through immersive experiences [34]. The 8-week self-administered program was compared to a sham VR intervention (neutral content without therapeutic components). Among 179 participants, the therapeutic VR group achieved a 1.41-point greater reduction in average pain intensity on a 0–10 scale compared to sham (95% CI: 0.49–2.34, *p* = 0.003). Notably, 66% of therapeutic VR users achieved ≥30% pain reduction compared to 41% in sham group (*p* = 0.001). Pain interference with daily activities, sleep quality, and mood all improved significantly more with therapeutic VR. Benefits persisted at 3-month follow-up, suggesting durable treatment effects. These findings supported FDA authorization of RelieVRx as a digital therapeutic for chronic low back pain in 2021 [77].

Virtual mind–body programs delivered via telehealth platforms have demonstrated effectiveness. Tankha et al. randomized 320 patients with CLBP to either virtual yoga classes (live-streamed group sessions twice weekly for 12 weeks) or education control [78]. The yoga intervention resulted in greater improvements in Roland–Morris Disability Questionnaire scores (−3.1 vs. −1.2, *p* < 0.001) and pain intensity (−1.9 vs. −0.6, *p* < 0.001). Interestingly, perceived social support from group participation correlated strongly with outcomes (r = 0.54, *p* < 0.001), highlighting the potential value of incorporating social elements into digital interventions. While RelieVRx has received FDA authorization as a prescription digital therapeutic for chronic low back pain, other interventions such as virtual mind–body programs and sensor-guided exercise platforms remain investigational, with promising results but pending formal regulatory evaluation.

#### 4.2.2. Adolescent Idiopathic Scoliosis

Scoliosis-specific exercise therapy represents a cornerstone of conservative AIS management but requires specialized training and consistent performance of complex exercises. A randomized clinical trial by Yuan et al. compared digitally supervised remote exercise programs to standard in-person therapy in 102 adolescents with AIS (Cobb angles 20–40°) [79]. The digital program used motion capture technology to assess exercise performance, provided real-time corrective feedback, and enabled remote supervision by trained therapists through video review and feedback.

After 6 months, the digital group achieved significantly greater improvements in Cobb angle compared to traditional therapy (mean reduction −3.7° vs. −1.9°, *p* = 0.014), with 41% of digital group patients improving by ≥5° versus 22% of traditional group patients (*p* = 0.041). Quality of life scores improved more with digital intervention (SRS-22 total score change +0.42 vs. +0.21, *p* = 0.008). Exercise adherence tracked through app logging averaged 4.8 sessions per week in the digital group compared to patient-reported 3.2 sessions weekly in traditional therapy (*p* < 0.001). Importantly, family satisfaction was higher with digital therapy due to reduced time commitment for traveling to appointments and greater flexibility in scheduling exercise sessions around school and activities. Although clinical outcomes are promising, these digitally supervised exercise platforms remain investigational, and no regulatory authorization has yet been granted for scoliosis-specific digital therapeutics.

### 4.3. Lower Extremity Applications

#### 4.3.1. Total Knee Arthroplasty

TKA represents an ideal application for DTxs given the high procedure volumes, critical importance of rehabilitation for functional outcomes, and substantial variability in access to quality physiotherapy services. The multicenter VERITAS randomized controlled trial provided landmark evidence for telerehabilitation effectiveness [35]. This large trial randomized 306 patients recovering from TKA to either virtual in-home exercise therapy or traditional outpatient clinic-based physiotherapy. The virtual intervention included video-guided exercise sessions, wearable activity trackers, and weekly video consultations with physical therapists.

At 12 weeks post-surgery, virtual rehabilitation proved non-inferior to traditional care across all primary and secondary functional outcomes. Knee injury and Osteoarthritis Outcome Scores (KOOS) improved by 42.3 points in the virtual group versus 43.1 points with traditional therapy (difference 0.8, 95% CI: −3.2 to 4.8), comfortably within the prespecified non-inferiority margin of 5 points. Knee flexion range of motion reached 115° in both groups (virtual 115.3° ± 12.4° vs. traditional 114.8° ± 13.1°, *p* = 0.72). Timed Up and Go test performance, 6 min walk distance, and patient satisfaction metrics all demonstrated equivalence between groups.

Importantly, the virtual intervention provided several advantages: patients reported significantly higher convenience scores (8.9/10 vs. 6.2/10, *p* < 0.001), completion rates were higher for virtual sessions (87% vs. 79%, *p* = 0.032), and per-patient costs were 41% lower ($2340 vs. $3960) when accounting for transportation, facility overhead, and time costs. Notably, adverse event rates were identical (4.6% vs. 4.9%, *p* = 0.91), with no difference in emergency department visits or hospital readmissions, demonstrating the safety of remote management.

Evidence from previously published systematic reviews and meta-analyses also reflects similar overall findings across broader patient populations [16], including the work of Tsang et al., who analyzed 18 randomized controlled trials encompassing 1256 patients and found that telerehabilitation after TKA yields statistically equivalent pain outcomes (standardized mean difference −0.12, 95% CI: −0.31 to 0.07) and functional outcomes (SMD 0.08, 95% CI: −0.15 to 0.31) compared to conventional rehabilitation [61]. Patient satisfaction ratings were marginally higher for telerehabilitation (mean difference 0.34/10, 95% CI: 0.12 to 0.56, *p* = 0.003), while travel burden and indirect costs were substantially reduced.

Wearable sensor-guided programs offer additional benefits through objective monitoring and automated feedback. Yang et al. described a randomized trial protocol for 120 patients using smart knee braces with embedded IMU sensors tracking knee flexion angles, extension lag, gait parameters, and daily activity levels [36]. Preliminary data showed that real-time feedback on achieving flexion milestones improved adherence to stretching exercises by 38% and reduced development of postoperative stiffness requiring manipulation under anesthesia from 4.2% to 1.3% (*p* = 0.048). King et al. reported similar benefits using wearable sensor systems providing biofeedback on gait symmetry and loading patterns, demonstrating 30% faster normalization of gait mechanics and improved quadriceps strength recovery [80].

#### 4.3.2. Anterior Cruciate Ligament Reconstruction

ACL reconstruction rehabilitation demands careful progression through multiple phases over 6–12 months, making it an ideal candidate for technology-enhanced monitoring and guidance. App- and sensor-based digital platforms are increasingly used to monitor recovery, track functional milestones, and assess psychological readiness for return to sport.

Gardner et al. surveyed 312 physical therapists specializing in ACL rehabilitation about telerehabilitation adoption and perceived barriers [81]. While 78% reported using some form of digital tool, comprehensive integration remained limited. Therapists identified monitoring quality of movement remotely (cited by 67%) and assessing psychological readiness (58%) as primary challenges. Digital platforms incorporating validated return-to-sport testing batteries showed promise, with one pilot study of 64 athletes demonstrating that app-guided progression criteria reduced variation in return-to-sport timing (standard deviation 3.2 weeks vs. 6.8 weeks with traditional care, *p* = 0.001) and improved consistency in meeting objective criteria before clearance.

A systematic review and meta-analysis by Li et al. examined virtual reality technology in ACL rehabilitation across 12 studies encompassing 487 patients [37]. VR-enhanced rehabilitation demonstrated small-to-moderate benefits for functional outcomes (SMD 0.34, 95% CI: 0.12–0.56) and psychological readiness (SMD 0.42, 95% CI: 0.18–0.66). However, high-quality randomized controlled trial evidence demonstrating superior return-to-sport rates or reduced reinjury remains limited, with most existing studies showing proof-of-concept rather than definitive clinical superiority. This represents an important area for future rigorous research.

### 4.4. Fracture Care and Nonunion Prevention

Tibial shaft fractures represent a common traumatic injury with significant nonunion risk (5–10% of cases) requiring extended healing monitoring. Warmerdam et al. conducted innovative studies using instrumented insoles for long-term gait monitoring following tibial fracture fixation [38]. In a prospective cohort of 42 patients, continuous insole-based monitoring captured gait parameters during daily activities over 12–16 weeks post-surgery. A recent narrative review on fracture management reported that telemedicine-based follow-up can enhance access, streamline postoperative monitoring, and facilitate early detection of complications, further supporting the relevance of digital approaches in this population [82].

The research identified specific gait signatures predictive of nonunion risk as early as 6 weeks post-surgery [38]. Patients who ultimately developed nonunion demonstrated persistent gait asymmetries (affected limb loading < 65% of contralateral limb), reduced step length (mean 42 cm vs. 53 cm in successful healers, *p* = 0.003), and altered temporal gait parameters (increased stance phase duration). Machine learning models analyzing these early gait patterns predicted nonunion with 82% accuracy (95% CI: 71–89%), sensitivity of 76%, and specificity of 84%. These findings suggest potential for early intervention strategies, though definitive evidence that digital monitoring-triggered interventions reduce nonunion rates requires prospective validation trials.

A concise overview of the evidence strength and technological readiness is summarized in Table 2.

## 5. Regulatory, Economic, and Implementation Considerations

### 5.1. Global Regulatory Landscape

The regulatory framework for musculoskeletal DTx varies significantly across jurisdictions, reflecting different approaches to balancing innovation encouragement with patient safety protection [22]. Understanding these regulatory pathways is essential for developers seeking market access and clinicians evaluating evidence quality.

#### 5.1.1. United States FDA Framework

In the United States, the Food and Drug Administration (FDA) classifies digital therapeutics as Software as a Medical Device (SaMD), applying risk-based regulatory frameworks [83]. The FDA’s Digital Health Innovation Action Plan, updated in 2020, established several pathways:

510(k) Premarket Notification: Most musculoskeletal DTx qualify for this pathway, requiring demonstration of substantial equivalence to previously cleared predicates. The process typically requires 3–6 months and costs $100,000–$300,000. Clinical data requirements vary based on predicate similarity, with many clearances achieved using bench testing and usability studies rather than randomized controlled trials.

De Novo Classification: Novel devices without appropriate predicates can pursue de novo pathways. This route requires more comprehensive evidence but establishes new device classifications that become predicates for subsequent 510(k) submissions. Review timelines extend to 6–12 months with costs of $300,000–$500,000.

Pre-Cert Program: The FDA’s Software Pre-Certification pilot program enables expedited review for developers demonstrating strong quality systems and culture of safety [84]. Five companies were selected for this pilot, which emphasizes organizational excellence over product-by-product review.

Enforcement Discretion: Low-risk wellness applications and general health trackers without specific treatment claims may qualify for enforcement discretion, avoiding premarket review requirements. However, the boundary between wellness and medical claims requires careful consideration.

#### 5.1.2. European Union Medical Device Regulation

The European Medical Device Regulation (MDR) 2017/745, which replaced the Medical Device Directive in May 2021, governs approval processes for DTx in EU markets [83]. Key requirements include:

CE Marking: Digital therapeutics must obtain CE marking through conformity assessment by Notified Bodies. Classification depends on intended purpose and risk level, with most musculoskeletal DTx falling into Class IIa or IIb categories requiring moderate-to-substantial clinical evidence.

Clinical Evaluation Reports: Comprehensive clinical evaluation reports (CERs) must demonstrate safety and performance through clinical data, whether from clinical investigations, published literature, or equivalent device data [23]. The rigor required has increased substantially under MDR compared to previous directives.

Post-Market Surveillance: Manufacturers must establish robust post-market surveillance systems, tracking device performance through Post-Market Surveillance Plans (PMSP) and Periodic Safety Update Reports (PSUR).

Unique Device Identification: All devices require UDI codes enabling traceability throughout the supply chain and integration with EUDAMED, the European database on medical devices.

#### 5.1.3. Asia-Pacific Regulatory Systems

Japan PMDA: The Pharmaceuticals and Medical Devices Agency categorizes digital therapeutics based on risk, with most requiring approval as “program medical devices.” The SAKIGAKE designation system provides accelerated pathways for innovative devices addressing unmet needs, offering priority consultation, expedited review (6 months vs. 12 months standard), and extended post-approval pricing protection [85].

Korea MFDS: The Ministry of Food and Drug Safety established dedicated digital healthcare pathways in 2019, recognizing software as medical devices with streamlined review processes for AI-based systems [86]. Clinical trial requirements can be reduced for devices with strong real-world evidence from foreign markets.

Australia TGA: The Therapeutic Goods Administration applies risk-based frameworks similar to FDA, with most musculoskeletal DTx classified as Class IIa or IIb requiring TGA registration and evidence of safety and performance.

#### 5.1.4. Data Privacy and Cybersecurity Requirements

Beyond device-specific regulations, musculoskeletal DTx must comply with comprehensive data protection frameworks:

HIPAA (United States): The Health Insurance Portability and Accountability Act requires strict protection of Protected Health Information (PHI) through technical safeguards (encryption, access controls), administrative safeguards (policies, training), and physical safeguards (facility security) [87]. Business Associate Agreements (BAAs) must govern data sharing with third parties [88].

GDPR (European Union): The General Data Protection Regulation establishes stringent requirements including explicit consent mechanisms, data minimization principles, right to erasure, data portability, and breach notification within 72 hours [89]. Cross-border data transfers require adequacy decisions or standard contractual clauses.

Cybersecurity Standards: The FDA’s cybersecurity guidance requires threat modeling, software bills of materials (SBOM), vulnerability management processes, and coordinated disclosure mechanisms [84]. ISO 27001 information security management certification (ISO/IEC 27001:2022) is increasingly expected by healthcare systems and payers [90].

### 5.2. Health Economics and Reimbursement

#### 5.2.1. Cost-Effectiveness Evidence

Musculoskeletal DTx offer substantial potential for cost savings through multiple mechanisms. Published economic evaluations have reported cost benefits across multiple domains:

Hospital Readmission Reduction: Digital monitoring enables early detection of complications before they escalate to emergency department visits or readmissions. Studies report 20–30% reductions in 90-day readmission rates following major orthopedic procedures when patients use digital therapeutics with automated alert systems [91]. At average readmission costs of $15,000–$25,000, this translates to savings of $3000–$7500 per high-risk patient.

Physiotherapy Visit Optimization: Traditional rehabilitation typically requires 12–24 in-person physiotherapy visits over 12 weeks at costs of $75–$150 per session ($900–$3600 total). Digital therapeutics can reduce in-person visits by 30–40% while maintaining equivalent outcomes, generating savings of $270–$1440 per patient [92]. A recent propensity-matched claims analysis by Napoleone et al. demonstrated that virtual physical therapy reduced total healthcare costs by $1103 per episode of care while improving patient satisfaction and access [92].

Surgical Revision Avoidance: Enhanced monitoring and adherence support may reduce revision surgery rates by 15–25% through earlier complication detection and better protocol compliance [93]. Given revision procedure costs of $30,000–$50,000, even modest relative risk reductions yield substantial absolute savings when applied across large patient populations.

Productivity and Indirect Costs: Hayes et al. performed economic evaluation of the REFORM randomized trial, demonstrating that remotely delivered physiotherapy for musculoskeletal conditions achieved cost savings of AUD $110 per patient for the health system and AUD $141 per patient in out-of-pocket expenses [93]. When societal perspectives incorporating lost productivity were considered, digital rehabilitation generated AUD $389 per patient in total savings through faster return to work and reduced caregiver burden.

Quality-Adjusted Life Years: Cost-effectiveness analyses using quality-adjusted life year (QALY) metrics demonstrate favorable incremental cost-effectiveness ratios. A Markov model analysis of digital therapeutics for chronic low back pain estimated incremental cost-effectiveness ratio of $12,400 per QALY gained compared to usual care, well below commonly cited willingness-to-pay thresholds of $50,000–$100,000 per QALY [94].

#### 5.2.2. Reimbursement Models and Coverage Policies

Despite promising cost-effectiveness evidence, sustainable reimbursement models remain underdeveloped in most healthcare systems, representing a critical barrier to widespread adoption.

Germany’s DiGA Program: Germany’s Digital Health Applications (DiGA) program, launched in 2020, provides a successful model with standardized reimbursement pathways [95]. Manufacturers can obtain provisional DiGA listing based on CE marking and plausible positive care effects, enabling 12 months of reimbursed market access during which rigorous clinical evidence is generated. Permanent listing requires demonstrating medical benefit or patient-relevant care improvements through comparative studies. The program has achieved remarkable success with 89% approval rate for submitted applications and growing catalog of reimbursed digital therapeutics covering multiple specialties including musculoskeletal care. Reimbursement rates are negotiated between manufacturers and statutory health insurance funds, typically ranging from €120–€500 for 90-day treatment courses.

United States Medicare and Medicaid: The Centers for Medicare & Medicaid Services (CMS) has begun covering select digital therapeutics through multiple mechanisms [96]. Temporary billing codes for remote therapeutic monitoring (RTM) established in 2022 enable reimbursement for device setup ($19), device supply ($55/month), and provider time reviewing data ($50 per 20 min). However, coverage remains limited and billing requires significant administrative burden. The CMS Innovation Center has launched several pilot programs testing bundled payment models that include digital therapeutics as components of comprehensive care pathways.

Private Payer Coverage: Commercial insurance coverage varies widely, with some payers establishing medical policy criteria for specific digital therapeutics while others maintain case-by-case prior authorization processes. Value-based contracting arrangements where reimbursement depends on demonstrated outcomes represent an emerging model aligned with digital therapeutics’ strong data generation capabilities.

Direct-to-Consumer and Employer Models: Some digital therapeutics companies pursue direct-to-consumer revenue streams or partnerships with self-insured employers seeking cost-management tools. Monthly subscription fees typically range from $39–$199, potentially more affordable than traditional physical therapy copays for many patients.

### 5.3. Implementation Challenges and Solutions

Successful clinical implementation requires addressing multiple interconnected challenges spanning technical, organizational, and human factors domains [97].

#### 5.3.1. Clinical Workflow Integration

Healthcare delivery organizations face significant challenges integrating digital therapeutics into existing clinical workflows without increasing clinician burden [98]. Common barriers include the following:

Time Constraints: Clinicians already facing productivity pressures cannot absorb substantial additional time reviewing digital therapeutic data. Solutions involve automated data summarization, exception-based reporting that highlights only concerning patterns requiring attention, and integration with existing EHR review workflows rather than separate platform logins.

EHR Integration: Seamless bidirectional data exchange between digital therapeutics platforms and electronic health records remains technically challenging due to varying EHR systems, limited standardization of rehabilitation-specific data elements, and institutional information technology governance processes. Health Level 7 (HL7) Fast Healthcare Interoperability Resources (FHIR) standards offer promising frameworks but require substantial implementation effort.

Training Requirements: Clinicians need education on interpreting digital therapeutic data, adjusting protocols based on remote monitoring insights, and communicating with patients about technology use. Effective training programs combine self-paced online modules, hands-on simulation exercises, and ongoing consultation support during initial implementation phases.

Reimbursement Complexity: Navigating billing codes, documentation requirements, and prior authorization processes for digital therapeutics adds administrative burden. Practice management support including billing specialists knowledgeable about digital health codes and standardized documentation templates can mitigate this barrier.

#### 5.3.2. Patient Engagement Strategies

Sustaining patient engagement over extended rehabilitation periods (often 12–24 weeks) requires sophisticated behavioral design [99,100]:

Gamification Elements: Points, badges, achievement levels, and progress visualizations leverage intrinsic and extrinsic motivation. Studies show gamified interventions increase exercise adherence by 35–45% compared to non-gamified digital programs. Effective implementations provide variable reward schedules, social comparison features (leaderboards or community challenges), and personalized goal-setting tied to functional outcomes meaningful to individuals.

Social Support Features: Peer support forums, family involvement tools, and group challenges create social accountability and normalization of rehabilitation challenges. Moderated online communities supervised by healthcare professionals can provide peer mentoring while ensuring medically accurate information exchange.

Personalization and Autonomy: Allowing patients input into exercise selection, scheduling flexibility, and pace of progression increases perceived autonomy and treatment alliance. Adaptive systems that respond to patient preferences while maintaining clinical effectiveness represent an optimal balance.

Push Notification Strategies: Timely reminders increase exercise completion but require careful design to avoid notification fatigue. Evidence-based approaches use variable timing, personalized messaging reflecting individual barriers, and reduction in frequency as habits solidify. Machine learning models can optimize notification timing based on individual response patterns.

Progress Visualization: Clear, interpretable displays of improvement trajectories help patients recognize progress that may feel incremental day-to-day. Comparative benchmarks showing performance relative to typical recovery curves provide context and motivation.

#### 5.3.3. Technical Infrastructure Requirements

Robust technical infrastructure enables reliable service delivery [101]:

Connectivity Considerations: Many patients, particularly in rural areas or lower-income communities, face limited broadband access or mobile data constraints. DTxs should function with intermittent connectivity, synchronize data when networks are available, and minimize data transmission requirements through efficient compression and edge processing.

Device Compatibility: Supporting diverse device ecosystems (iOS and Android phones and tablets, various operating system versions, older hardware) requires extensive testing and maintenance. Progressive web applications offer cross-platform compatibility but may sacrifice some native app capabilities.

Cybersecurity Measures: Healthcare delivery organizations require comprehensive security assessments including penetration testing, vulnerability scanning, and security architecture reviews before approving digital therapeutics for clinical use. Compliance with frameworks like HITRUST Common Security Framework demonstrates robust security posture.

Scalability and Reliability: Cloud infrastructure must handle varying loads as patient populations grow, maintain 99.9%+ uptime commitments, and implement disaster recovery capabilities ensuring data persistence and service continuity during failures.

#### 5.3.4. Evidence Generation and Quality Improvement

Continuous evidence generation supports ongoing refinement and demonstrates value to stakeholders [102]:

Real-World Evidence Collection: Unlike traditional medical devices with limited post-market data collection, digital therapeutics enable comprehensive tracking of usage patterns, outcomes, and patient characteristics at scale. Registry-based studies leveraging aggregated de-identified data can generate insights unattainable through conventional research methods.

Rapid Iteration Cycles: Digital therapeutics’ software nature enables continuous improvement through iterative updates. However, this creates regulatory challenges around when changes require new submissions versus qualify as maintenance updates. The FDA’s AI/ML-based SaMD guidance proposes predetermined change control plans allowing specified modifications without new submissions.

Pragmatic Clinical Trials: Embedded randomization and automated outcome collection enable pragmatic trials assessing effectiveness in real-world clinical settings with minimal disruption to care delivery. These studies provide more generalizable evidence than traditional highly controlled efficacy trials.

Quality Dashboards: Healthcare systems implementing digital therapeutics should establish quality metrics including adoption rates, adherence patterns, outcome improvements, safety signals, and user satisfaction scores. Regular review of these metrics enables identification of implementation challenges and continuous refinement.

## 6. Future Directions and Emerging Innovations

### 6.1. Digital Twin Technology and Precision Rehabilitation

Digital twin technology represents perhaps the most transformative emerging capability for musculoskeletal rehabilitation, enabling truly personalized treatment planning through computational simulation [98,103].

#### 6.1.1. Technical Architecture of Digital Twins

Musculoskeletal digital twins integrate multiple data streams into comprehensive computational models [104]: high-resolution medical imaging (MRI, CT) providing anatomical geometry; finite-element models simulating tissue mechanical properties and loading responses; surgical parameters documenting procedure-specific details like graft placement, implant positioning, or osteotomy corrections; wearable sensor data capturing real-world biomechanics and activity patterns; patient-reported outcomes tracking symptoms and functional status; and physiological monitoring including markers of inflammation, healing, and metabolic status.

Advanced computational techniques enable these models to simulate rehabilitation scenarios. Musculoskeletal modeling software like OpenSim or AnyBody creates physics-based simulations of joint mechanics, muscle forces, and tissue loading during various activities [105]. Machine learning models trained on thousands of similar cases predict healing trajectories and complication risks based on patient-specific parameters. Optimization algorithms identify rehabilitation protocols maximizing functional recovery while minimizing complication risks [106].

#### 6.1.2. Clinical Applications of Digital Twins

For rotator cuff repair, digital twins could integrate preoperative MRI defining tear size and muscle quality, arthroscopic video capturing repair construct, postoperative imaging confirming healing, and wearable sensor data quantifying actual tissue loading during daily activities [107]. The model simulates various rehabilitation intensities, predicting which protocols optimize healing while minimizing retear risk for that specific patient. Early research suggests that such models can identify patients benefiting from accelerated protocols versus those requiring extended protection phases with 78–82% accuracy.

For chronic conditions like osteoarthritis, digital twins longitudinally track disease progression, predicting trajectories years in advance based on current state and intervention responses. This enables proactive intervention timing and personalized prevention strategies.

#### 6.1.3. Barriers and Development Pathway

Substantial technical, regulatory, and implementation challenges must be overcome before digital twins achieve widespread clinical deployment. Computational costs currently limit real-time simulation capabilities, though advances in edge computing and model compression techniques are rapidly improving feasibility. Model validation requires extensive prospective studies demonstrating that simulation-based predictions actually correspond to real-world outcomes, necessitating large registries tracking simulated versus observed results. Regulatory frameworks must evolve to address continuously learning models that update based on accumulating data. Clinician education and interface design need development to make complex simulations interpretable and actionable by practicing orthopedic surgeons rather than requiring computational expertise. Despite these challenges, pilot implementations in academic medical centers are demonstrating proof-of-concept, with commercialization anticipated within 5–7 years. Nevertheless, current evidence for digital twins remains preliminary, and their long-term clinical impact will require rigorous prospective validation.

### 6.2. Advanced Predictive Analytics and Complication Prevention

Machine learning models analyzing comprehensive datasets will increasingly enable proactive rather than reactive complication management [108,109].

#### 6.2.1. Early Warning Systems

Real-time risk stratification models analyze streaming data from multiple sources to identify subtle deviation patterns preceding clinical complications [108]. For example, following ACL reconstruction, models integrating daily pain scores, activity levels, swelling assessed via smartphone photography, and knee laxity measured with handheld devices can predict excessive inflammation or early graft failure 2–3 weeks before clinical presentation. Triggered by crossing risk thresholds, automated alerts prompt clinician review and potential intervention such as adjusting activity restrictions, prescribing anti-inflammatory medications, or scheduling urgent evaluation.

Similar systems for fracture healing analyze serial gait data from wearable insoles, identifying loading pattern abnormalities predictive of nonunion, malunion, or hardware failure. Prospective validation studies are testing whether such early warnings combined with proactive interventions (e.g., dynamization of fixation, bone stimulation, activity modification) actually reduce complication rates compared to standard episodic monitoring.

#### 6.2.2. Adherence Prediction and Personalized Support

Machine learning models predict individual patients’ adherence trajectories based on early-phase behavior patterns, demographic factors, psychological variables, and social determinants of health. Patients identified as high-risk for nonadherence receive enhanced support interventions such as increased coaching contact, motivational interviewing, barrier-specific problem-solving assistance, or peer mentorship connections [110]. Preliminary studies suggest such targeted approaches improve overall population adherence by 20–25% while efficiently allocating limited support resources to patients most likely to benefit [111].

Natural language processing applied to patient-reported symptom narratives and chat interactions with digital health coaches can identify concerning themes (catastrophizing, depression, social isolation, financial strain) triggering appropriate referrals to behavioral health, social work, or financial counseling services. Nonetheless, these predictive analytics systems remain early-stage technologies, and their clinical reliability will depend on broader validation across diverse patient populations.

### 6.3. Multimodal Integration and Systems Medicine Approach

Future musculoskeletal DTx will transcend purely biomechanical and functional metrics, integrating diverse data streams encompassing biological, psychological, and social determinants of rehabilitation success [85].

#### 6.3.1. Biological Markers and -Omics Integration

Emerging research identifies molecular biomarkers predicting rehabilitation responses and complication risks. For example, genetic polymorphisms affecting collagen synthesis, inflammatory response, and pain sensitivity influence tendon and ligament healing rates. Proteomic analyses of synovial fluid or serum identify molecular signatures associated with osteoarthritis progression trajectories. As point-of-care biomarker testing becomes more accessible and affordable, integrating such biological data with digital therapeutics enables precision rehabilitation stratification.

Metabolomic profiles reflecting nutritional status, metabolic health, and inflammatory states provide actionable targets for adjunctive interventions supporting tissue healing [112]. Digital platforms could integrate nutritional guidance, sleep optimization strategies, and stress management based on individual metabolic profiles rather than generic recommendations.

#### 6.3.2. Psychological and Social Determinants

Psychological factors profoundly influence musculoskeletal rehabilitation outcomes, with depression, anxiety, kinesiophobia, and catastrophizing predicting poor recovery across conditions [113]. Future DTx will seamlessly integrate psychological assessment and intervention, delivering cognitive-behavioral strategies, mindfulness training, and motivational support personalized to individual psychological profiles.

Social determinants including socioeconomic status, social support networks, occupation, and environmental factors substantially impact rehabilitation success. Machine learning models incorporating these variables provide more accurate outcome predictions and identify patients requiring enhanced psychosocial support services. Integration with community resources, social services, and peer support networks creates comprehensive wraparound care addressing multiple barriers to recovery.

#### 6.3.3. Integration with Robotic and Assistive Technologies

Convergence of digital therapeutics with robotic rehabilitation devices and assistive technologies creates synergistic ecosystems. Robotic exoskeletons and rehabilitation devices capture high-fidelity biomechanical data during supervised sessions while digital therapeutics guide home exercise programs during unsupervised periods. Data integration creates seamless longitudinal tracking spanning all rehabilitation contexts.

Similarly, integration with smart implants containing embedded sensors will enable unprecedented insight into in vivo loading, implant stability, and tissue healing. For example, instrumented joint replacements measuring contact forces, range of motion, and activity patterns throughout the implant lifespan could inform rehabilitation progression and detect early loosening or instability before clinical symptoms emerge.

## 7. Discussion

This review summarizes current knowledge and emerging trends in musculoskeletal digital therapeutics, demonstrating substantial promise while acknowledging important limitations and knowledge gaps requiring ongoing research. Accordingly, the long-term durability and real-world effectiveness of many DTx modalities remain to be established.

### 7.1. Synthesis of Clinical Evidence

The clinical evidence base for musculoskeletal DTx has expanded substantially over the past five years, progressing from small pilot studies to adequately powered randomized controlled trials across multiple anatomical regions and patient populations. Several consistent patterns emerge from this literature. While the review encompasses multiple digital technologies, this work intentionally focused its discussion on clinical applicability and validation evidence in musculoskeletal rehabilitation—particularly in shoulder, spine, and lower extremity disorders—to avoid superficial coverage of peripheral technologies. This review emphasized domains with a sufficient volume of high-quality clinical trials and standardized functional outcome measures to ensure comparability across studies and reduce bias introduced by under-researched or heterogeneous conditions.

Non-Inferiority for Primary Outcomes: The strongest evidence demonstrates that properly designed digital therapeutics achieve outcomes comparable to traditional in-person rehabilitation across most functional measures. The VERITAS trial for TKA rehabilitation, multiple RCTs for rotator cuff repair rehabilitation, and published reviews across conditions consistently show non-inferiority for primary efficacy endpoints. This equivalence evidence is critically important, establishing that remote digital delivery does not compromise clinical outcomes when replacing in-person care.

Superiority for Secondary Benefits: While functional outcomes show equivalence, digital therapeutics demonstrate advantages in secondary domains including patient convenience, accessibility, adherence, and potentially cost-effectiveness. These benefits have substantial public health significance, as improved access and adherence translate to broader population-level impact even when individual-level efficacy is equivalent.

Across the available trials, roughly two-thirds of studies evaluating telerehabilitation or sensor-guided rehabilitation reported outcomes comparable to conventional care, whereas evidence for VR- and AI-based systems remains smaller in volume and more variable in methodological quality.

Heterogeneity in Implementation: Substantial variability exists in technological sophistication, clinical integration, and outcomes across different digital therapeutic implementations. This heterogeneity partially reflects the field’s relative immaturity, with ongoing iteration and optimization of designs. It also highlights that technology alone is insufficient—successful implementations require careful attention to clinical protocols, patient support, workflow integration, and continuous quality improvement.

Evidence Gaps: Important evidence gaps persist, requiring rigorous research. Long-term outcomes beyond 12–24 months remain understudied for most applications. Cost-effectiveness analyses with rigorous prospective economic data collection are limited. Comparative effectiveness research directly contrasting different digital therapeutic approaches is scarce. Subgroup analyses identifying which patients benefit most from digital versus traditional care are insufficiently powered in most trials. Pragmatic implementation science research examining real-world adoption, sustainability, and scaling in diverse healthcare settings requires expansion. In addition, the overall strength of evidence remains constrained by the limited number of large, multicenter randomized controlled trials and the methodological variability across published studies. Considerable heterogeneity in study design, intervention delivery, outcome definitions, and follow-up duration further limits the generalizability and comparability of reported results.

The wide variability in methodological rigor—including inconsistent randomization procedures, heterogeneous comparator interventions, small sample sizes, and short follow-up durations—further limits comparability across studies and introduces uncertainty regarding the magnitude and durability of reported effects. In addition, potential publication bias may inflate perceived treatment benefits, particularly in areas where industry-affiliated studies predominate and negative or neutral findings are less likely to be published.

Overall, the available evidence suggests generally supportive findings regarding the clinical effectiveness of digital therapeutics in musculoskeletal rehabilitation. A formal evidence-grading process was not performed, the qualitative appraisal of study quality, risk-of-bias levels, and consistency of findings (see Table 1). Provides a transparent reflection of the overall strength of evidence. While the overall evidence base is encouraging, the strength of conclusions is tempered by methodological variability and moderate risk of bias observed across studies. These factors underscore that current findings should be interpreted with caution, emphasizing the need for larger, well-controlled trials to confirm the observed benefits of musculoskeletal digital therapeutics.

In summary, the maturity of clinical evidence differs across musculoskeletal domains. The most robust data are available for post-TKA and rotator cuff repair rehabilitation, supported by multiple randomized controlled trials and meta-analyses confirming non-inferiority to conventional in-person therapy. However, the overall body of evidence remains heterogeneous, with variable methodological quality and limited long-term validation. Spine rehabilitation and chronic low back pain show moderate evidence, with several controlled studies supporting feasibility but limited long-term validation. In contrast, ACL reconstruction, fracture rehabilitation, and hand conditions remain in early investigational phases, where available studies are small, heterogeneous, and lack standardized outcome measures. These distinctions clarify the relative maturity of evidence across clinical domains and highlight priority areas for future research.

In addition, potential publication bias must be considered, as studies reporting positive or favorable outcomes are more likely to be published. A considerable proportion of available evidence also originates from industry-sponsored or technology-affiliated trials, which may influence study design, analysis, or interpretation. Independent, investigator-initiated studies and transparent reporting practices are essential to ensure balanced and reproducible evidence for musculoskeletal digital therapeutics.

Overall, the included studies demonstrated generally low-to-moderate risk of bias, most commonly related to small sample sizes, limited blinding, and short follow-up durations, which were considered when interpreting the findings.

### 7.2. Implementation Science Perspectives

Translation from efficacy trials to routine clinical practice faces substantial barriers that explain the disconnect between promising research findings and limited widespread adoption. Implementation science frameworks like RE-AIM (Reach, Effectiveness, Adoption, Implementation, Maintenance) provide useful lenses for analyzing these challenges [108]:

Reach: Digital therapeutics potentially expand beyond traditional service delivery, particularly for geographically isolated or mobility-limited populations. However, digital divides based on age, socioeconomic status, technological literacy, and infrastructure access risk excluding vulnerable populations most likely to face rehabilitation access barriers. Equity-focused implementation strategies including device lending programs, digital navigator support, and hybrid digital-traditional models can mitigate these concerns. In addition, global differences in digital infrastructure and technology affordability create substantial disparities in accessibility. Broadband availability, device ownership, and healthcare IT capacity differ markedly across regions, resulting in consistently lower access to digital therapeutics in low-resource or rural settings unless supported through coordinated policy initiatives, infrastructure investment, and system designs that operate reliably under constrained connectivity.

Adoption: Clinician adoption depends on perceived advantage relative to existing practice, compatibility with workflows and values, simplicity of use, and observability of benefits [109]. Many digital therapeutics require substantial behavior change from clinicians accustomed to hands-on treatment models, creating adoption barriers. Champion-driven implementation, peer influence networks, and demonstrating value through local data accelerate adoption.

Implementation Fidelity: Even when adopted, implementations often deviate from intended protocols, potentially compromising effectiveness [110]. Common fidelity challenges include inadequate patient onboarding, inconsistent clinician review of digital therapeutic data, technical troubleshooting difficulties, and drift from prescribed protocols over time. Ongoing training, technical support infrastructure, and fidelity monitoring systems support high-quality implementation.

Maintenance and Sustainability: Initial enthusiasm often wanes over time, particularly without sustainable reimbursement and demonstrated value. Embedding digital therapeutics within organizational performance metrics, quality improvement initiatives, and financial models promotes long-term sustainability.

### 7.3. Balancing Innovation and Evidence

Musculoskeletal digital therapeutics operate in tension between rapid technological innovation and healthcare’s appropriate emphasis on robust evidence. This tension manifests in several ways:

Regulatory Frameworks: Current regulatory approaches based on fixed devices requiring substantial premarket evidence do not align perfectly with continuously evolving software. The FDA’s predetermined change control plans for AI/ML-based SaMD represent progress, but implementation details remain under development. Balancing innovation enablement with patient safety protection requires ongoing regulatory evolution.

It should also be acknowledged that numerous commercially available applications have entered the market without undergoing formal regulatory clearance, while others developed within research projects have not progressed to commercialization. This mismatch between technological proliferation and regulatory oversight highlights the need for harmonized pathways to ensure both innovation and patient safety.

Evidence Standards: Applying pharmaceutical-style evidence requirements (large RCTs, extended follow-up, multiple replication studies) to rapidly evolving digital technologies creates challenges. By the time such evidence accumulates, technology may have advanced substantially. Adaptive trial designs, real-world evidence methodologies, and pragmatic trials offer potential solutions but require greater acceptance by regulators, payers, and clinical communities.

Clinical Integration: Healthcare organizations face challenges determining which digital therapeutics to adopt given limited resources, competing demands, and variable evidence maturity. Health technology assessment frameworks specifically designed for digital therapeutics, emphasizing iterative evaluation and real-world performance monitoring, can guide decision-making.

### 7.4. Future Research Priorities

Several research priorities warrant emphasis to advance the field:

1. Comparative Effectiveness Research: Head-to-head comparisons of different digital therapeutic approaches identifying optimal technological features and clinical protocols.
2. Personalization Algorithms: Development and validation of algorithms matching specific digital therapeutic characteristics to individual patient profiles.
3. Implementation Science Studies: Detailed evaluation of implementation strategies, identifying factors supporting successful adoption, fidelity, and sustainability across diverse settings.
4. Economic Evaluations: Expanded cost-effectiveness analyses from societal perspectives with extended time horizons.
5. Digital Biomarker Validation: Establishing relationships between digital metrics and clinically meaningful outcomes.
6. Health Equity Research: Understanding and addressing digital divide impacts; developing strategies ensuring equitable access and outcomes.
7. Long-Term Outcomes: Extended follow-up assessing durability of treatment effects and potential disease-modification impacts.

## 8. Conclusions

Musculoskeletal DTx are transforming orthopedic rehabilitation by integrating AI, wearable sensors, cloud analytics, and immersive interfaces to deliver personalized, scalable, and accessible care. Clinical trials across multiple subspecialties confirm non-inferior functional outcomes compared to conventional therapy, with added benefits including improved adherence (40% to 75%), functional score gains (12–15 points), and reduced complications (30–50%). However, their widespread clinical adoption remains contingent upon overcoming key challenges related to regulatory approval, reimbursement, equitable access, and digital literacy. Although emerging innovations such as digital twins, predictive analytics, and multimodal data integration hold substantial promise, their long-term clinical effectiveness and economic value require confirmation through robust longitudinal studies and rigorous cost-effectiveness evaluations. Sustained collaboration among clinicians, researchers, developers, policymakers, and patients will be essential to translating technological promise into safe, evidence-based, and equitable musculoskeletal care. In practical terms, clinicians and health systems should view DTx as complementary tools that can enhance rehabilitation capacity, improve monitoring efficiency, and support more personalized care delivery when integrated thoughtfully into existing workflows.

## Figures and Tables

**Table 1:** Characteristics of Studies included in the Review.

Domain	Authors (Year)	Study Type	Study Group	Technology Type	Sample Size	Follow-Up Period	Main Outcomes
Shoulder/upper extremity	Correia et al. (2022) [31]	RCT	Post-ARCR rehabilitation	Wearable sensor + mobile application	50	12 months	Function: Constant-Murley: 82.4 vs. 80.1 early; 12 M CMS + 5.7 Function: QuickDASH: 8.2 vs. 14.7 (Δ = 6.5) Resource use: In-person visits ↓ 40%
	Shim et al. (2023) [32]	RCT	Post-ARCR rehabilitation	Augmented Reality (AR)	105	6 months	ROM: FF 156° vs. 142°, ER 52° vs. 46° Pain: VAS ↓ by 1.3 Adherence: 81% vs. 58%
Spine	Shebib et al. (2019) [33]	RCT	Chronic low back pain	Motion analysis + CBT + tele-coaching	140	3 months	Pain (NRS): −3.2 vs. −1.1 ODI: −8.2 points Opioid use ↓ 40%
	Garcia et al. (2021) [34]	RCT	Chronic low back pain	Virtual Reality (VR)	179	2 months	Pain (NRS): −1.4 vs. sham FDA-authorized DTx
Lower Extremity	Prvu Bettger et al. (2020) [35]	Multicenter RCT	Post-TKA rehabilitation	Tele-rehabilitation	306	12 months	KOOS: +42.3 vs. +43.1 (non-inferior) ROM: similar (115°) Cost ↓ 41%
	Yang et al. (2023) [36]	RCT (pilot)	Post-TKA rehabilitation	Smart knee brace	120	6 months	Adherence ↑ 38% MUA rate: 4.2% → 1.3%
	Li et al. (2025) [37]	Meta-analysis	Post-ACL reconstruction	Virtual Reality (VR)	487 (12 studies)	Varied (4–24 weeks across studies)	Small-to-moderate functional gains (SMD 0.34)
Fracture/Bone Healing	Warmerdam et al. (2025) [38]	Prospective cohort	Tibial shaft fracture fixation	Smart insole + AI gait analysis	42	6 weeks	Predicted nonunion at 6 weeks (AUC 0.82)

Summary of key clinical studies evaluating DTx in musculoskeletal rehabilitation across orthopedic subspecialties. All included interventions met the Digital Therapeutics Alliance (DTA) definition of digital therapeutics (DTx). ARCR, arthroscopic rotator cuff repair; AR, augmented reality; AI, artificial intelligence; CBT, cognitive behavioral therapy; DTx, digital therapeutics; ROM, range of motion; VAS, visual analog scale; NRS, numeric rating scale; ODI, Oswestry Disability Index; KOOS, Knee injury and Osteoarthritis Outcome Score; CMS, Constant-Murley Score; TKA, total knee arthroplasty; ACL, anterior cruciate ligament; SMD, standardized mean difference; AUC, area under the curve; RCT, randomized controlled trial.

**Table 2:** Summary of Evidence Level and Technological Readiness of Musculoskeletal Digital Therapeutics.

Clinical Domain	Representative Applications	Level of Evidence (LoE)	Technological Readiness Level (TRL)	Regulatory Status	Key References
Shoulder Rehabilitation	Sensor-guided home exercise, tele-supervised physiotherapy	Moderate–High (multiple RCTs showing non-inferiority)	7–8 (clinical validation stage)	Not yet authorized	[31,32,71,72,73,74,75]
Spine (CLBP)	Multimodal DTx, VR-based pain programs (RelieVRx)	High (multiple RCTs and meta-analyses)	8–9 (approved DTx in market)	FDA-authorized (RelieVRx)	[76,77,78]
Spine (AIS)	Digitally supervised scoliosis-specific exercise therapy	Moderate (single RCT)	5–6 (pilot–feasibility stage)	Investigational	[79]
Knee (TKA)	Telerehabilitation platforms, wearable sensor-guided programs	High (large multicenter RCTs and meta-analyses)	7–8 (clinical validation stage)	Not yet authorized	[16,35,36,80]
ACL Reconstruction	App- or sensor-based remote monitoring, VR-enhanced rehab	Moderate (pilot and meta-analyses)	6–7 (early clinical validation)	Not yet authorized	[37,81]
Fracture Care	Smart insole–based gait monitoring and ML-based risk prediction	Low–Moderate (prospective cohort data)	5–6 (proof-of-concept stage)	Not yet authorized	[38]

LoE = Level of Evidence; TRL = Technological Readiness Level; DTx = Digital Therapeutics; RCT = Randomized Controlled Trial; CLBP = Chronic Low Back Pain; AIS = Adolescent Idiopathic Scoliosis; TKA = Total Knee Arthroplasty; ACL = Anterior Cruciate Ligament. LoE: High = multicenter RCT/meta-analysis or regulatory-approved DTx; Moderate = RCTs with limited size or consistency; Low = observational or pilot studies. TRL: 5–6 = pilot/feasibility; 7–8 = clinical validation; 9 = approved/marketed.

## Data Availability

No new data were created.

## Abbreviations

The following abbreviations are used in this manuscript:

MSD	musculoskeletal disorders
TKA	total knee arthroplasty
THA	total hip arthroplasty
DTx	digital therapeutics
AI	artificial intelligence
IMU	inertial measurement unit
HIPPA	health insurance portability and accountability act
GDPR	general data protection regulation
VR/AR	virtual reality/augmented reality
RCT	randomized controlled trial
ARCR	arthroscopic rotator cuff repair
CLBP	chronic low back pain
FDA	food and drug administration
SaMD	software as a medical device

## Appendix A. Representative Search Strings

PubMed: (“digital therapeutic*” OR “digital health” OR “mHealth” OR “eHealth” OR “telerehabilitation” OR “tele-rehabilitation” OR “virtual rehabilitation” OR “remote monitoring”) AND (“musculoskeletal” OR “orthopedic” OR “physiotherapy” OR “physical therapy” OR “rehabilitation”) AND (“artificial intelligence” OR “machine learning” OR “wearable sensor” OR “mobile application*” OR “smartphone”)

Embase: (‘digital health’/exp OR ‘eHealth’ OR ‘telerehabilitation’ OR ‘remote monitoring’) AND (‘musculoskeletal disease’/exp OR ‘orthopedic rehabilitation’) AND (‘machine learning’/exp OR ‘artificial intelligence’/exp OR ‘wearable device’)

Web of Science: TS = (“digital therapeutic*” OR “digital health” OR “telerehabilitation” OR “remote monitoring”) AND TS = (“musculoskeletal” OR “orthopedic” OR “physiotherapy” OR “rehabilitation”)

AND TS = (“artificial intelligence” OR “machine learning” OR “wearable sensor”)

IEEE Xplore: (“digital health” OR “motion analysis” OR “wearable sensors”) AND (“musculoskeletal” OR “rehabilitation”) AND (“machine learning” OR “artificial intelligence”)

## Appendix B. Technical Architectures and Validation Summaries

**Table A1:** Summary of Computational Architectures and Validation Results for Computer Vision-based Motion Analysis Systems.

Component	Technical Description	Representative Algorithms/Models	Validation Metrics	Key References
Pose Estimation	Markerless detection of joint and body landmarks using RGB or depth images. Extracts 2D/3D skeletal coordinates in real time (30–60 fps).	OpenPose; DeepLabCut; MediaPipe; HRNet	Mean joint localization error < 10 mm; ICC > 0.90 for shoulder/hip ROM	[39,40,41,42,43,44]
Biomechanical Modeling	Converts pose data to joint angles, velocities, and accelerations using kinematic models.	Inverse kinematics solver; Newton–Euler equations	Angular accuracy ± 2–5°; concurrent validity r = 0.88–0.94 vs. motion lab	[42,43,44]
Exercise Classification	Recognizes rehabilitation exercise type and movement phase.	CNN + LSTM hybrids; transformer-based temporal networks	Classification accuracy 92–97%; correlation r = 0.78–0.89 with therapist ratings	[46,114,115]
Movement Quality Assessment	Compares patient motion to reference patterns using temporal alignment.	Dynamic time warping; recurrent neural networks	Detection sensitivity > 90% for deviations; reproducibility ICC > 0.85	[46,114,115]
Feedback Generation	Provides automated corrective cues based on deviation analysis.	Rule-based systems; AI adaptive feedback engines	Real-time feedback latency < 100 ms	[45,46,114]
System Implementation	Multi-camera or depth-sensor configuration eliminating occlusion; smartphone or tablet-based deployment.	Depth sensing (Intel RealSense; Azure Kinect); mobile AR frameworks	Cross-platform latency < 50 ms; user satisfaction > 90% in pilot studies	[39,45]

Detailed performance data and model configurations are derived from the studies cited. ROM = Range of motion; ICC = Intraclass correlation coefficient; CNN = Convolutional neural network; LSTM = Long short-term memory.

**Table A2:** Summary of Major Wearable Sensor Modalities, Signal Processing Components, and Validation Outcomes Relevant to Musculoskeletal Digital Therapeutics.

Component	Technical Description	Representative Devices/Algorithms	Validation Metrics /Clinical Findings	Key References
Inertial Measurement Units (IMUs)	Tri-axial accelerometers; gyroscopes and magnetometers embedded in smart bands; patches or insoles; sensor-fusion algorithms estimate joint angles and limb kinematics (100–1000 Hz sampling).	Commercial IMUs (e.g., Shimmer, Xsens, MetaMotion); Kalman-filter and complementary-filter fusion methods.	Angular accuracy ± 2°; detects 5% step-length asymmetry and 10 ms stance-phase timing difference; excellent reliability for ROM monitoring.	[48,49]
Surface Electromyography (EMG)	Records muscle electrical activity for neuromuscular control and fatigue assessment; single or multi-channel (64–256 electrodes) arrays.	Dry or gel electrode wearables (MyoWare, Delsys Trigno); HD-EMG mapping software.	Signal-to-noise ratio > 20 dB; inter-trial ICC > 0.90; detects fatigue onset within < 5 s lag; supports biofeedback training.	[50,51,52]
Smart Insoles	Force-sensitive resistors or capacitive pressure sensors across plantar surface to measure weight-bearing and gait symmetry.	Moticon Science, Plantiga, custom AI gait analysis models.	Adherence to weight-bearing protocols ↑ 40–60%; excessive-load violations ↓ 35–45%; AUC = 0.82 for predicting non-union risk.	[53,54]
Upper-Extremity Wearables	Smart watches, arm bands, and finger sensors quantify range of motion and daily limb use; differentiate actual use from capacity.	Apple Watch, Fitbit Sense, custom IMU bands.	Detect arm-elevation frequency correlated with clinic ROM scores (r = 0.84); use rate ↑ 30% in tele-rehab programs.	[55]
Data Integration & Feedback	Sensor data uploaded to mobile apps or cloud dashboards for adherence monitoring and real-time alerts.	Mobile DTx platforms (e.g., Sword Health, Kaia); adaptive feedback algorithms.	Real-time feedback latency < 150 ms; patient adherence ↑ 25–35% vs. standard care.	[47,54,55]

IMU = Inertial measurement unit; EMG = Electromyography; ICC = Intraclass correlation coefficient; ROM = Range of motion; AUC = Area under the receiver operating characteristic curve.

**Table A3:** Summary of Cloud-based and AI-driven System Components supporting Musculoskeletal Digital Therapeutics.

Component	Technical Description	Representative Algorithms/Platforms	Validation Metrics	Key References
Cloud Infrastructure	Centralized data management for musculoskeletal DTx; secure cloud storage and remote access	AES-256 encryption, SSL/TLS protocols, redundant data centers (≥99.99% uptime)	Network latency < 150 ms; zero data loss under failover	[56,57,58]
Edge Computing Layer	Local preprocessing and artifact filtering to reduce network load	Real-time compression and noise filtering modules	Data volume reduction 30–40% prior to upload	[57]
Clinician Dashboard/Visualization	Aggregates rehabilitation data (ROM, adherence, pain, outcomes) for decision support	Multi-stream integration dashboards, interactive visualization engines	User satisfaction > 90%; alert precision 85–92%	[58]
Machine Learning Analytics	Predicts functional recovery and personalizes rehabilitation	Supervised, reinforcement, and deep neural models (CNN, LSTM, RL agents)	Accuracy 82–90%; AUC-ROC 0.75–0.88 for retear risk	[59,60,61,62,63,64]
Adaptive Control Algorithms	Adjust exercise parameters dynamically according to patient response	Feedback-driven adaptive control systems	Functional improvement +25–40% vs. static protocols	[63]
Digital Twin Models	Simulate individualized rehabilitation trajectories before clinical implementation	Multimodal integration of imaging, biomechanical, and sensor data	Predictive accuracy > 85%; simulation reliability ICC = 0.88	[65,66]

DTx = Digital therapeutics; AES = Advanced encryption standard; SSL/TLS = Secure sockets layer/Transport layer security; ROM = Range of motion; CNN = Convolutional neural network; LSTM = Long short-term memory; RL = Reinforcement learning; AUC-ROC = Area under the receiver operating characteristic curve; ICC = Intraclass correlation coefficient.

**Table A4:** Summary of Virtual and Augmented Reality Systems used in Musculoskeletal Digital Therapeutics.

Component	Technical Description	Representative Devices/Algorithms	Validation Metrics	Key References
Immersive VR Rehabilitation Systems	Full virtual environments providing task-oriented or gamified exercise training	Head-mounted displays (HTC Vive, Oculus Quest); game-based exercise software	Adherence ↑ 40–60%; functional gain superior to control (*p* < 0.05)	[67,68,69,70]
Augmented Reality Rehabilitation	Overlays digital visual guides on real movements for trajectory and alignment feedback	Microsoft HoloLens; ARKit/ARCore motion-tracking frameworks	Motion accuracy error < 5°; user satisfaction > 90%	[31,71]
VR-Based Pain Management	Immersive scenarios modulating pain via distraction and cognitive engagement	Interactive VR modules incorporating CBT and mindfulness training	Pain reduction 30–50%; effect size d = 0.5–0.8	[72,73]
Haptic and Multisensory Systems	Provides tactile and proprioceptive feedback to enhance immersion and motor learning	Haptic gloves, force-feedback controllers, wearable vibration devices	Reaction latency < 20 ms; improved motor accuracy 10–15%	[74]
Social and Collaborative VR Platforms	Enables multi-user rehabilitation sessions and remote therapist supervision	Networked VR environments with shared avatars and voice feedback	Adherence ↑ > 20%; session engagement > 85%	[74]

VR = Virtual reality; AR = Augmented reality; ARKit = Augmented Reality Kit; ARCore = Augmented Reality Core; CBT = Cognitive behavioral therapy.
